# Supplementary material for: Gendered narratives and cultural shifts: A qualitative study on decadal changes in community alcohol consumption
Source: PLoS One. 2024 Dec 2;19(12):e0309378. doi: 10.1371/journal.pone.0309378 (PMC11611190; doi:10.1371/journal.pone.0309378)
Supplement: Attachment — Submitted filename: with comments PONE-D-23-38278_R1_reviewer.pdf [file pone.0309378.s002.pdf]

PLOS ONE

Gendered narratives and cultural shifts: a qualitative study on decadal changes in community alcohol consumption

--Manuscript Draft--

**Manuscript Number:**	PONE-D-23-38278R1
**Article Type:**	Research Article
**Full Title:**	Gendered narratives and cultural shifts: a qualitative study on decadal changes in community alcohol consumption
**Short Title:**	Gendered narratives and cultural shifts
**Corresponding Author:**	Natchana Bhutasang<br>Shinawatra University - Pathumthani Campus: Metharath University<br>Pathum Thani, THAILAND
**Keywords:**	Keywords: Sabah community, Gendered alcohol perceptions, Alcohol-related interventions
**Abstract:**	This qualitative investigation delves into the evolving dynamics of alcohol consumption within the Sabah community, historically rooted in traditional practices but now facing the pressures of modernization and global influences. Based on in-depth analysis from the western coast of Sabah, the study uncovered a multi-faceted interplay of factors: gender roles, personal values, family influences, and societal perceptions. While alcohol's cultural significance remains intact, changing gender dynamics reflect broader societal shifts. Additionally, family roles, especially parental behaviors, have a complex influence on offspring's alcohol habits. Alarmingly, issues like domestic violence, linked to alcohol, surfaced with varying definitions between genders. Local community regulations, especially village customary laws, offer promising solutions. However, participants highlighted a glaring lack of efficient health services. The study underscores the need for community-centric solutions and further research that captures the diverse nuances within the broader Sabah community.
**Order of Authors:**	Ming Gui Tan
	Walton Wider
	Nicholas Tze Ping Pang
	Helen Benedict Lasimbang
	Wendy Diana Shoesmith
	Leilei Jiang
	Natchana Bhutasang
**Response to Reviewers:**	Reviewer 1:<br>My only comment is that you review the form of citation of the document, since they must be surnames and in some citations they have names, just verify according to the editorial guidelines. It is a good article and of great importance for theoretical bases of this problem.<br><br>Response: Thank you for the comments. We have revised accordingly.<br><br>Reviewer 2:<br>This manuscript on gendered narratives and cultural shifts in alcohol consumption in the Sabah community has some potential. However, I found it wasn't guided by a clear research question<br><br>Response: We have made amendments and came up with a stronger research question.<br><br>I'm also not sure this is the most appropriate journal for it (perhaps a journal more oriented sociological/cultural studies and substance use)

Powered by Editorial Manager® and ProduXion Manager® from Aries Systems Corporation

Response: We feel that PLOS One is a suitable journal, as alcohol use is a very interdimensional and intertextual disorder that cuts across medicine, psychology, sociology and gender, hence we do not want to limit the scope by sending it to a very specific journal, but rather to a broad based journal.

I found the abstract difficult to follow – it jumped around without contextualising Sabah or providing a clear research question. More detail is needed here, and I would suggest a more structured abstract based off the introduction, methods, results and conclusion might be helpful.

Response: We have made necessary amendments according to this comment.

The structure of the paper needs to be outlined much earlier in the introduction, which I think will help. While the information is there, the current structure doesn't always flow very coherently between topics and ideas

Response: We have restructured the introduction to present the outline clearly.

You state the "study seeks to examine the trajectory of alcohol consumption within the Sabah community over the years" but I'm not sure you can do this with a study of only one timepoint. Perhaps it might be better to phrase this as reflections of cultural and gender shifts in alcohol consumption in Sabah

Response: This has been amended.

The studies raised in the literature need elaboration – what did the studies do and why did you draw on them? Some of the studies seem to be focused on Sabah but others are drawn from Malaysia more broadly. What can/can't these studies tell us? Where is the research gap you are filling?

Response: We've revised the paper to provide more details on cited studies, clarified their distinctions, and emphasized our contribution to filling the research gap

The methods are generally ok, but going through the COREQ checklist may help clarify some things. Importantly, the "Data Analysis" section is very underdone. How was the data "processed manually"? Was thematic analysis used? If so, how was it used? I'm also a little unsure about the use of ChatGPT as a tool to assist with analysis given the potential for bias/inaccuracies

Response: We reviewed the COREQ checklist to ensure clarity in our methods. We acknowledged the need for further detail in the "Data Analysis" section and elaborated on how the data was processed manually through thematic analysis. Regarding the use of ChatGPT, we understood your concerns about bias and inaccuracies and provided additional justification for its inclusion or considered alternative methods.

I found many of the themes too brief to provide substantial analysis and interpretation. Lots of words were used up writing out both translations of quotes (authors should decide if this is worth the wordcount used) and I didn't feel like the themes told enough of a coherent story. I would consider collapsing themes if/where possible, but also using a reformulating the themes so they don't read like lists of quotes from participants

Response: Thank you for your feedback. We have reviewed the themes and acknowledged the brevity you mentioned. We reconsidered the use of wordcount on translations of quotes and made adjustments. We collapsed themes where possible and reformulated them to ensure they provide a more coherent narrative, rather than reading like lists of participant quotes.

I felt the discussion spent a lot of time reviewing the literature, but didn't incorporate the findings to draw out comparisons/differences from previous research. A stronger discussion that draws on both relevant literature alongside your findings would provide clearer implications/conclusions for the paper.

Response: We have revised the discussion so that it is now more concise now and

	draws out comparisons/differences from previous research.
**Additional Information:**	
**Question**	**Response**
**Financial Disclosure**<br><br>Enter a financial disclosure statement that describes the sources of funding for the work included in this submission. Review the submission guidelines for detailed requirements. View published research articles from *PLOS ONE* for specific examples.<br><br>This statement is required for submission and **will appear in the published article** if the submission is accepted. Please make sure it is accurate.<br><br>**Funded studies**<br>Enter a statement with the following details:<br>• Initials of the authors who received each award<br>• Grant numbers awarded to each author<br>• The full name of each funder<br>• URL of each funder website<br>• Did the sponsors or funders play any role in the study design, data collection and analysis, decision to publish, or preparation of the manuscript?<br><br>Did you receive funding for this work?	The author(s) received no specific funding for this work.
**Competing Interests**<br><br>Use the instructions below to enter a competing interest statement for this submission. On behalf of all authors, disclose any competing interests that could be perceived to bias this work—acknowledging all financial support and any other relevant financial or non-financial competing interests.<br><br>This statement is required for submission and **will appear in the published article** if the submission is accepted. Please make sure it is accurate and that any funding	NO authors have competing interests<br>Enter: The authors have declared that no competing interests exist.

sources listed in your Funding Information later in the submission form are also declared in your Financial Disclosure statement.<br><br>View published research articles from *PLOS ONE* for specific examples.<br><br>**NO authors have competing interests**<br><br>Enter: *The authors have declared that no competing interests exist.*<br><br>**Authors with competing interests**<br><br>Enter competing interest details beginning with this statement:<br><br>*I have read the journal's policy and the authors of this manuscript have the following competing interests: [insert competing interests here]*<br><br>* typeset	
**Ethics Statement**<br><br>Enter an ethics statement for this submission. This statement is required if the study involved:<br><br>• Human participants<br>• Human specimens or tissue<br>• Vertebrate animals or cephalopods<br>• Vertebrate embryos or tissues<br>• Field research<br><br>Write "N/A" if the submission does not require an ethics statement.<br><br>General guidance is provided below. Consult the submission guidelines for detailed instructions. **Make sure that all information entered here is included in the Methods section of the manuscript.**	Medical Research Ethics Committee, Universiti Malaysia Sabah<br>Code: JKEtika 3/20 (6)<br>Written consent was obtained from all participants

**Format for specific study types**<br><br>**Human Subject Research (involving human participants and/or tissue)**<br>• Give the name of the institutional review board or ethics committee that approved the study<br>• Include the approval number and/or a statement indicating approval of this research<br>• Indicate the form of consent obtained (written/oral) or the reason that consent was not obtained (e.g. the data were analyzed anonymously)<br><br>**Animal Research (involving vertebrate animals, embryos or tissues)**<br>• Provide the name of the Institutional Animal Care and Use Committee (IACUC) or other relevant ethics board that reviewed the study protocol, and indicate whether they approved this research or granted a formal waiver of ethical approval<br>• Include an approval number if one was obtained<br>• If the study involved *non-human primates*, add *additional details* about animal welfare and steps taken to ameliorate suffering<br>• If anesthesia, euthanasia, or any kind of animal sacrifice is part of the study, include briefly which substances and/or methods were applied<br><br>**Field Research**<br><br>Include the following details if this study involves the collection of plant, animal, or other materials from a natural setting:<br>• Field permit number<br>• Name of the institution or relevant body that granted permission	
**Data Availability**<br><br>Authors are required to make all data underlying the findings described fully available, without restriction, and from the time of publication. PLOS allows rare exceptions to address legal and ethical concerns. See the PLOS Data Policy and FAQ for detailed information.	Yes - all data are fully available without restriction

A Data Availability Statement describing where the data can be found is required at submission. Your answers to this question constitute the Data Availability Statement and **will be published in the article**, if accepted.<br><br>**Important:** Stating 'data available on request from the author' is not sufficient. If your data are only available upon request, select 'No' for the first question and explain your exceptional situation in the text box.<br><br>Do the authors confirm that all data underlying the findings described in their manuscript are fully available without restriction?	
**Describe where the data may be found in full sentences. If you are copying our sample text, replace any instances of XXX with the appropriate details.**<br><br>• If the data are **held or will be held in a public repository**, include URLs, accession numbers or DOIs. If this information will only be available after acceptance, indicate this by ticking the box below. For example: *All XXX files are available from the XXX database (accession number(s) XXX, XXX.*).<br>• If the data are all contained **within the manuscript and/or Supporting Information files**, enter the following: *All relevant data are within the manuscript and its Supporting Information files.*<br>• If neither of these applies but you are able to provide **details of access elsewhere**, with or without limitations, please do so. For example:<br><br>*Data cannot be shared publicly because of [XXX]. Data are available from the XXX Institutional Data Access / Ethics Committee (contact via XXX) for researchers who meet the criteria for access to confidential data.*<br><br>*The data underlying the results presented in the study are available from (include the name of the third party*	https://doi.org/10.5281/zenodo.10813061

and contact information or URL).<br>• This text is appropriate if the data are owned by a third party and authors do not have permission to share the data.<br><br>* typeset	
Additional data availability information:	Tick here if your circumstances are not covered by the questions above and you need the journal’s help to make your data available.

Powered by Editorial Manager® and ProduXion Manager® from Aries Systems Corporation

Manuscript
Click here to access/download;Manuscript;Qualitative Analsysis Revision 26032024 (untracked).docx

Gendered narratives and cultural shifts: A qualitative study on decadal changes in community alcohol consumption

Ming Gui Tan[1], Walton Wider[2], Nicholas Tze Ping Pang[3], Helen Benedict Lasimbang[3], Wendy Diana Shoesmith[3,4], Leilei Jiang[5], & Natchana Bhutasang[6,*]

[1]Department of Psychiatry and Psychological Health, Hospital Universiti Malaysia Sabah, Jalan UMS 88400 Kota Kinabalu Malaysia
[2]Faculty of Business and Communications, INTI International University, Nilai 71800, Negeri Sembilan, Malaysia
[3]Faculty of Medicine and Health Sciences, Universiti Malaysia Sabah, Jalan UMS 88400 Kota Kinabalu Malaysia
[4]Derbyshire Healthcare NHS Foundation Trust, Trust Headquarters Kingsway Hospital Derby Derbyshire DE22 3LZ
[5]Faculty of Education and Liberal Arts, INTI International University, Nilai 71800, Negeri Sembilan, Malaysia
[6]Faculty of Health Sciences, Shinawatra University, Pathum Thani 12160, Thailand
*Corresponding Author: natchana.b@mru.ac.th

Abstract

This study explores the impact of gender and cultural transformations on alcohol consumption within Sabah, Malaysia, over the last decade. Employing a qualitative approach, we conducted focus group interviews with members of the native community to delve into its evolving relationship with alcohol. The study reveals a significant shift in drinking habits. Historically, alcohol was mostly consumed by men, but now more women are drinking, changing long-standing gender roles related to alcohol use. Children often copy their parents' drinking behaviors, and with alcohol being more easily available, more people are drinking than before. Our findings also expose the complex consequences of alcohol use, which extend to health concerns, familial tension, and economic hardship. Despite the entrenched cultural status of alcohol, these negative outcomes are exacerbated by a lack of supportive healthcare services. In light of these insights, the study suggests the need for intervention plans that respect the cultural background of Sabah while tackling the current issues of alcohol misuse. The research adds to the wider conversation about managing alcohol in different cultural settings and highlights the importance of creating informed policies and health programs that are tailored to the specific needs of the community.

Keywords: Sabah community, Gendered alcohol perceptions, Alcohol-related interventions

Introduction

Sabah, a state that exhibits notable cultural and economic differences compared to West Malaysia, is recognized as one of the leading regions in the country with a significantly elevated prevalence of alcohol consumption (1). Sabah, situated on the island of Borneo, showcases distinctive socio-cultural dynamics notably shaped by the Kadazandusun community (2). Within this context, it is worth noting the significant prevalence of alcohol misuse (3, 4). In Sabah's traditional celebrations, alcohol consumption has been deeply ingrained throughout

history (5). This is especially true during the harvest festival known as the Kaamatan festival. This annual harvest ceremony holds great significance among the Kadazandusun community (6). In this context, it is observed that the consumption of traditional home-brewed alcoholic beverages such as Tapai and Montoku holds significance, as they have been historically associated with various ceremonial occasions including the New Year, Christmas, birthdays, and routine social gatherings (7-9).

Nevertheless, the influence of alcohol surpasses mere ceremonial practices. Alcohol has been found to have significant problematic implications in various aspects of Sabah, including domestic violence, absenteeism from work, motor accidents, and physical confrontations (4, 10). Approximately 23.6% of alcohol consumption in Sabah can be classified as "risky," indicating the significant nature of this matter (11). Paradoxically, the complex interplay of cultural elements presents a significant challenge in addressing the issue of alcohol misuse (12). The inherent conflict between traditional values and the consequences of intoxication creates a reluctance among individuals to openly acknowledge and confront their alcohol-related problems (10).

The consumption of alcohol is closely associated with cultural practices, particularly within indigenous communities (1). However, there is a lack of clear distinction between traditional usage and excessive consumption. For example, the Kadazan-Dusun community is acknowledged for its distinctive patterns of alcohol consumption and ceremonies involving alcoholic beverages (13). However, they also place significant emphasis on the principles of moderation, societal engagement, and spiritual obligations (2). The presence of contrasting elements within the cultural perception of alcohol underscores the need for a comprehensive and nuanced comprehension.

Our study explores how alcohol consumption in Sabah has changed due to shifts in gender roles and culture over the past decade. Initially, we provide a literature review to give background on the role of alcohol in the history and culture of Sabah. We then detail our methods, focusing on how we used focus group discussions and thematic analysis to understand alcohol use. The findings section reveals the influence of gender and culture on how people in Sabah view and use alcohol. This research adds to the ongoing conversation about alcohol consumption in the region. Our goal is to offer valuable insights and lay down a strong foundation for future work to create well-informed interventions.

Literature Review

Sociocultural, economic, and demographic factors are closely linked to the patterns of alcohol consumption in Sabah, Malaysia. In order to enhance our comprehension of these patterns, it is crucial to provide further details on the particular studies mentioned and their impacts on the field. Joseph et al. (8) highlighted the contrasting effects of alcohol by examining its influence on various aspects such as health, behavior, social dynamics, economy, and psychology. This study was utilized for its extensive analysis of the impacts of alcohol, which serves as a basis for comprehending the complex aspects of alcohol consumption in Sabah. The authors demonstrated that moderate alcohol consumption can yield beneficial effects on both individuals and communities. Nevertheless, the act of consuming an excessive amount of alcohol in an irregular manner results in substantial adverse outcomes, affecting not only the individuals who consume the alcohol but also their families and the broader

community. This differentiation emphasizes the subtle and sophisticated approach required when contemplating the role of alcohol in society.

Alcohol plays a crucial role in the cultural and traditional practices of the Indigenous communities in Sabah, serving as an essential component deeply woven into their social structure. In their study, Abd Rashid et al. (3) specifically examined the indigenous communities of Sabah. They discovered a troubling increase in alcohol consumption, which was influenced by gender norms and religious beliefs. This study is crucial as it emphasizes the distinctive elements specific to Sabah, setting it apart from the wider

Malaysian contexts and emphasizing the need for focused research in this region. Shoesmith et al. (14) conducted a comprehensive analysis of the various economic factors related to alcohol consumption, with a specific focus on the effects of taxation and smuggling. This research is crucial for comprehending the economic dynamics that impact alcohol consumption patterns in Sabah, providing localized solutions to tackle these challenges. In their analysis of alcohol consumption across Malaysia, Robert Lourdes et al. (1) and Mutalip et al. (11) broadened the scope to encompass demographic determinants, such as gender, age, education, employment, and smoking habits. These studies play a crucial role in identifying larger patterns and vulnerabilities, but their relevance to Sabah specifically requires careful examination of the local sociocultural context to ensure applicability. In addition, Manickam et al. (15) provided information regarding the frequency of alcohol consumption among Chinese students and Borneo natives, offering insights into the cultural foundations of alcohol consumption during ceremonies and social events. This study, in addition to the research conducted by Mutalip et al. (16) on the prevalence of binge drinking among the Bumiputera Sabah and Sarawak populations, highlights the importance of cultural factors in East Malaysia.

All together, these studies demonstrate the variety of factors influencing alcohol consumption patterns in Sabah and Malaysia at large. Nevertheless, the research deficiency exists in merging these discoveries to construct a thorough comprehension that is both culturally attuned and tailored to the distinct sociocultural, economic, and demographic conditions of Sabah. This study seeks to address this gap by integrating these observations into a coherent examination, pinpointing areas of consensus and discord in existing research, and explaining implications for policy and intervention approaches specifically designed for the Sabah context.

Methodology

Participants

The western coast is the focal point of the alcohol abuse epidemic due to cultural dynamics. Four zones in this region, namely Kota Kinabalu (KK), Kudat, Penampang, and Kiulu, became our target study areas, covering a vast geographical spread of 160km, thereby offering a diverse range of perspectives. The recruitment period started from 1 July 2020 to 31 August 2021.

One or more focus groups were formed in each target study area. A volunteer sampling method was utilised to recruit a group consisting of 6 to 7 study participants in each area. The youngest participant was 14 years old whereas the oldest was 81 years old. To incentivize participation, reimbursements were offered. Our study involved 7 focus group discussions, comprising 6 to 7 members each.

Procedure

Each discussion group was supervised by a research associate accompanied by a facilitator. Participants consisted of mixed genders. Group discussions were held in common gathering area of the community, such as a community hall. The facilitator's responsibilities included recruiting participants, recording the session, and transcribing the audio files. Upon beginning the session, the study's objectives and issues regarding confidentiality were clarified to the attendees. Subsequently, they were requested to provide their signatures on informed consent forms.

Interviews were primarily conducted in Bahasa Malaysia, the nation's official language. However, the local dialect was occasionally employed to facilitate a deeper comprehension. The interview of each focus group took about 1.5 hour and was based on the following prompt questions:

1. How has alcohol consumption in your community evolved over the past decade?
2. How do gender and culture affect the alcohol consumption in your community?

Once the interviews concluded, participants were granted reimbursements in diverse forms such as food supplies, T-shirts, and masks.

Reflexivity

The urban-centric perspective may limit researchers' insight into the cultural significance and accessibility of alcohol in the community. Additionally, the main researcher's psychiatric background introduces potential negative presumptions towards alcohol use, requiring careful consideration.

To address these challenges, researchers maintained reflective diaries to document and manage potential biases. Regular discussions with supervisors provided external perspectives, ensuring a more nuanced interpretation of the data. This reflexivity process aims to enhance the study's credibility by minimizing the impact of inherent biases on the qualitative analysis.

Ethical Considerations

During our qualitative research, we recognized participants might be hesitant to share due to concerns about community backlash and the worry of painting their community in a bad light. To address these concerns and ensure the accuracy of our data, we implemented strict measures to protect participants' identities. Instead of using names, we used alphabetical codes (A to G) and group numbers (1 to 7). Specific details about locations or other identifying information were intentionally left out. By taking these measures, we aimed to build trust and ensure participants felt comfortable sharing their genuine experiences.

Data Analysis

Initially, audio recordings from participant interviews were accurately transcribed verbatim. A third-party assistant with specialized training handled this transcription, aiming for both precision and an impartial reflection of participants' statements to minimize potential biases at the outset. Upon acquiring the transcribed data, our analysis proceeded through

manual thematic analysis. ChatGPT was employed to streamline the organization and management of data, ensuring efficient retrieval and categorization to support the research team's manual analysis.

Our analytical process began with a meticulous examination of the transcripts to uncover primary patterns or themes related to alcohol consumption and its community impact. The thematic analysis approach was systematic, incorporating several essential phases to guarantee its thoroughness and reliability.

During the code development phase, we generated a series of codes to categorize observations from the data. These codes emerged from recurring patterns and themes in the transcripts, particularly focusing on various aspects of alcohol consumption and its socio-cultural, economic, and demographic ramifications. Through iterative discussions within the research team, these initial codes were refined. This collaborative effort not only facilitated the identification of main themes but also ensured our analysis was grounded in the data and reflected the complexity of participants' experiences.

An expert review process was integral to evaluating the themes arising from our analysis. It involved soliciting feedback from seasoned researchers in the field and representatives of the studied community. By presenting our findings to these stakeholders during meetings, we could thoroughly vet our thematic conclusions, enhancing the credibility of our analysis. Furthermore, peer debriefing sessions served as an additional layer of scrutiny, providing vital oversight of the analytical process. These sessions enabled the research team to scrutinize and refine thematic conclusions, reinforcing the reliability and credibility of our findings.

Results

Based on all the responses gathered, four themes emerged.

The Cultural Backbone and Its Evolution

The consumption of alcohol in our community is deeply intertwined with traditions and changing perceptions. Historically, alcohol has played a pivotal role in celebrations and rites of passage, a sentiment echoed by a respondent who remarked on the inescapability of tradition, "...cannot escape tradition..." (B6). Another respondent highlighted the ceremonial importance of sharing the first alcoholic drink with parents, signifying a rite of passage that was once deeply respected. However, contemporary narratives suggest a shift from these traditional moorings. One respondent captured this evolution, stating, "It feels like the traditional meaning has been lost now. People used to drink to celebrate, but now it's more to run away from problems." (F1). This indicates a profound change in the role and significance of alcohol within the community—from a symbol of joy and communal bonding to a means of escape from personal and societal issues.

The societal upheavals brought on by the COVID-19 pandemic have further influenced alcohol consumption habits. A stark portrayal of this change was shared by a participant: "The use of alcohol has changed from the past because there was no COVID-19 pandemic before; people spent time going to work to earn a living. Now, because of the COVID-19 pandemic, many are out of work, many don't know what to do, and they drink alcohol to kill time." (F1) This reflection not only underscores the impact of the pandemic on daily life and employment

but also highlights the adaptive—or maladaptive—shifts in alcohol consumption as a coping mechanism. Amidst this evolving environment, researchers observed the emergence of distinct patterns of consumption, characterized by some individuals attempting to moderate their consumption while others engaging in excessive indulgence. Particularly concerning is the trend of underage drinking, a deviation from traditional norms potentially fuelled by generational influences. "However, a few of them who are not of legal drinking age have indeed been taking it secretly without their parents' knowledge." (G6), reveals the normalization of alcohol consumption among the youth, challenging the community's established norms and expectations.

The Double-Edged Sword of Visibility and Accessibility

The alcohol availability in our community has experienced substantial changes, as highlighted by a respondent's observation: "In this place, there are no obstacles or prohibitions to buying alcohol" (E2), illustrating the ease with which alcohol can now be purchased. This lack of restrictions has opened the door to various means of access, including the tradition of homebrewing. One participant shared their experience with localized production: "Here, we used to make our own alcohol using rice and medicine, and it had to be made for a week before it could be consumed" (A3), highlighting the ability in making homemade alcohol with everyday ingredients.

The journey from scarcity to abundance is further articulated by another respondent, who notes the stark contrast between past and present: "It used to be hard to find stores that sold alcohol. Now they are almost on every corner" (A2). This change points to a dramatic increase in the physical visibility of alcohol, making it a commonplace feature in the community's landscape. Echoing this sentiment, another adds, "Alcohol is cheaper and easier to find compared to ten years ago" (F7), indicating not only increased availability but also affordability over time. The ubiquity of alcohol is captured in the routine experiences of community members, as one person remarked, "...we often go into Speedmart 99. When we go in, we see it there." (E6). This statement reflects how alcohol has become a regular item on the shopping list, seamlessly integrated into daily life and consumer habits.

Navigating Social Influences and Evolving Gender Norms in Alcohol Consumption

The patterns of alcohol consumption in our community are greatly influenced by peer pressure and changing gender norms, which demonstrate the interaction of social constructs. One respondent encapsulated the peer pressure phenomenon among the youth, stating, "Sometimes, when consumed among teenagers, it is referred to as 'psycho', wanting to show oneself as someone strong" (G1). This insight reveals how, in some circles, alcohol consumption among teenagers is tied to displays of strength or bravado. The social aspect of drinking is further emphasized by another participant: "Moreover, if there are friends, they meet up and drink straight away. I'm the same, if initially I drink a little, over time it becomes until I'm drunk, and only after I've had enough do I sleep" (D3). Such statements underline the influence of peer gatherings on alcohol consumption patterns, suggesting a trend where socializing often escalates to excessive drinking.

A particularly noteworthy shift is observed in the gender dynamics of alcohol consumption. Historically dominated by men, the practice has seen increasing participation

from women. "I remember before only men drank. Now, I see many women also drinking" (A4), one respondent remarked, highlighting this changing trend. Furthermore, women's involvement in alcohol production, traditionally a male-dominated activity, signifies a notable shift towards gender equality in this realm: "Before, I thought only men could make alcohol at home. Now, I see many women also trying to make it themselves" (C4). Despite these advancements, societal judgments persist, with a discernible bias in how male and female drinkers are perceived: "There's a difference in our society. If a man drinks, people say he's strong. But if a woman drinks, they look down on her" (B4). This dichotomy suggests that while progress has been made, deep-seated cultural norms and expectations still influence the acceptance of female alcohol consumption.

Family influence also plays a pivotal role in shaping attitudes and behaviors toward alcohol. The narrative, "If parents drink, it's highly likely their children will also drink" (A2), points to the generational transmission of drinking habits, underscoring the impact of parental behavior on children. This notion of familial legacy in alcohol consumption is further supported by another participant, who observed, "...it's been from long ago, from parents, grandparents, they indeed drank. So we, our generation now, follow in their footsteps" (E3). Such observations highlight the significance of family in the continuum of alcohol consumption practices within the community.

The Multifaceted Impact of Alcohol on Life's Dimensions

The consumption of alcohol casts a wide net of repercussions, affecting various aspects of life such as health, relationships, work, and financial stability. The journey into alcohol use often begins with experimentation in youth, leading some to discover its counterproductive effects on personal problems. As one individual poignantly reflects, "I used to drink to forget my problems. But now I realize it only adds to the problems" (D2), this realization underscores the ineffectiveness of alcohol as a coping mechanism.

Health concerns associated with alcohol consumption are a significant worry for many, with individuals noting the detrimental effects on their well-being. "I try to stop drinking because I feel it affects my health" (D2), reveals the conscious decision to mitigate these health risks by reducing alcohol intake. The acknowledgment of alcohol's role in exacerbating health issues highlights the need for awareness and intervention in managing consumption. Beyond personal health, alcohol's ripple effects extend to the fabric of familial relationships, with some observing its capacity to strain bonds within the family unit. "I feel drinking alcohol can damage health and family relationships" (D7), encapsulates the broader social consequences of alcohol use, suggesting that its impact reaches beyond the individual to touch the lives of those around them. The workplace is not immune to the influences of alcohol, as evidenced by accounts of job losses attributed to drinking. "I know several people who lost their jobs because they got drunk at work" (F1), points to the severe implications of alcohol misuse in a professional setting, affecting not only individual careers but also organizational productivity and safety. Financial strain is another critical concern tied to alcohol consumption. The shift from spending on alcohol to prioritizing family finances, as shared by one respondent, "I used to spend a lot of money on alcohol. Now I try to save that money for my family" (D7), reflects a growing awareness of the economic toll of excessive drinking. This perspective shift underscores the recognition of the need to reallocate resources more responsibly.

Overall, the stories surrounding alcohol consumption demonstrate an interaction of negative consequences in terms of health, relationships, employment, and finances. These testimonies emphasize the difficulties presented by alcohol, prompting a more thorough analysis of its impact on society and individuals' lives. To tackle these concerns, a comprehensive strategy is needed that combines health education, support networks, and community involvement to navigate the complex effects of alcohol on various aspects of life.

Discussion

The discussion reveals a nuanced understanding of the evolving patterns of alcohol consumption within the Sabah community, particularly along its western coast, elucidating the intricate interplay of cultural, social, and health-related factors. Historically, alcohol has been deeply ingrained in the community's cultural fabric, playing a central role in ceremonies and celebrations, akin to many indigenous societies globally (17). Traditional alcoholic beverages like tapai, lihing, tumpung, and bahar have symbolized communal unity and ancestral customs, though their significance has evolved over time, with alcohol now serving as a coping mechanism for some individuals amidst life's challenges (18, 19).

The dynamics of alcohol consumption have undergone shifts, influenced by significant events such as the COVID-19 pandemic and the subsequent Movement Control Order (MCO) in Malaysia. While urban areas experienced a reduction in alcohol consumption due to restrictions on mass gatherings, rural regions witnessed a continuation of traditional drinking habits, indicating a geographical shift in consumption patterns (20). These changes reflect broader global trends where societal stressors drive individuals towards substance use (20, 21). Additionally, community-level availability and marketing of alcohol play a role in shaping consumption behaviors, particularly among adolescents (22).

The discussion also highlights the influence of age, urbanization, and beverage preferences on alcohol consumption. Older generations in rural areas tend to favor traditional homemade beverages, whereas younger demographics in urban settings lean towards commercially available alcoholic drinks (23). Youth involvement in alcohol consumption is concerning, with peer influence and societal pressures driving premature and potentially hazardous drinking habits (24). Furthermore, the evolving gender dynamics in alcohol consumption reveal a transition towards increased participation of women, reflecting broader shifts in gender roles, albeit with lingering gendered perceptions and societal judgments (3). Family dynamics and community regulations also shape alcohol-related behaviors, with parental attitudes towards alcohol influencing offspring in complex ways (25).

The detrimental effects of alcohol on health, relationships, and domestic violence underscore the need for comprehensive interventions, including educational counselling services on moderate alcohol use (26, 27). Despite the inadequacy of health services related to alcohol, initiatives driven by religious affiliations have dominated legislative discussions, indicating a gap in addressing core issues (27). Overall, the discussion emphasizes the multifaceted nature of alcohol consumption within the Sabah community and the need for tailored interventions to address its cultural, social, and health-related dimensions.

Limitations and Recommendations for Future Research

The research presented primarily focuses on the western coast of the Sabah community, which might not capture the entirety of practices and perspectives prevalent in other regions within Sabah. This geographical focus presents a limitation in understanding the full depth and breadth of alcohol consumption patterns within the broader Sabah community. Moreover, the data captured in the study is up to the present time, and given the fluid nature of societal perspectives and practices, the findings might not encapsulate potential future shifts.

Another significant consideration is the background of the researcher as a healthcare staff member. This may have introduced a potential bias in the way participants responded, as they might either consciously or subconsciously provide answers they deemed 'acceptable' or 'expected' within a healthcare context. This could particularly influence responses related to health outcomes, services, and perceptions of alcohol's effects.

Additionally, while the study emphasizes the historical and cultural importance of alcohol to the community, there might be limitations in the depth of exploration concerning specific rites, rituals, and traditions surrounding its use. Addressing the sensitive aspects of the research, especially around gender dynamics and the role of alcohol in domestic issues, might also have been influenced by reporting biases, especially in areas like domestic violence.

For a more comprehensive understanding of the relationship of the Sabah community with alcohol, it would be beneficial to expand the geographical scope of future studies. This would ensure a richer understanding by capturing diverse nuances within the entire community. A longitudinal approach, observing and noting changes over extended periods, could provide invaluable insights into the evolving patterns of alcohol consumption and its broader implications.

Given the potential bias introduced due to the researcher's background, future studies should consider involving a diverse team of researchers from various backgrounds, ensuring a more neutral data collection process. This could alleviate participants' concerns and encourage more candid responses.

A dedicated focus on the specific cultural and ceremonial uses of alcohol might offer a deeper dive into its traditional significance. Collaborating across disciplines with anthropologists, sociologists, and health professionals can paint a more holistic picture.

Alarming trends among the youth have been highlighted in the findings, pointing to a need for more in-depth studies targeting this demographic. Feedback on the inadequacy of health services related to alcohol since 2017 also indicates a gap that needs addressing. Evaluating these services in detail could highlight areas needing improvement.

Lastly, considering the effectiveness of localized strategies, like village customary laws, could pave the way for designing interventions that resonate with the community. Future research can thus address current limitations and provide a robust foundation to tackle the multifaceted challenges faced by the Sabah community in relation to alcohol.

Conclusion

The narratives from the Sabah community reveal a complex interplay in the dynamics of alcohol consumption. While its cultural significance remains undeniable, changing perceptions, evolving gender roles, and global events like the pandemic underscore the seismic

shift in the community's relationship with alcohol. As global influences intersect with local traditions, the emerging challenges are multifaceted and demand an intricate understanding of the evolving patterns and their foundational societal structures. Addressing these transitions necessitates a holistic approach that both honors the deeply embedded cultural roots and confronts the contemporary challenges. It is imperative to champion community-based solutions, elevate awareness, and untangle the intricate web of influences to effectively navigate this shifting landscape.

References

1. Robert Lourdes TG, Abd Hamid HA, Riyadzi MR, Rodzlan Hasani WS, Abdul Mutalip MH, Abdul Jabbar N, et al. Findings from a nationwide study on alcohol consumption patterns in an upper middle-income country. International Journal of Environmental Research and Public Health. 2022;19(14):8851.

2. Wider W, Suki NM, Lott ML, Nelson LJ, Low SK, Cosmas G. Examining criteria for adulthood among young people in sabah (East Malaysia). Journal of Adult Development. 2021;28(3):194-206.

3. Abd Rashid RB, Mohd Daud MNB, Guad RM, Gan SH, Wan Husin WAIB, Giloi N, et al. Prevalence and risk factors associated with alcohol consumption among indigenous people in Sabah Borneo Island. Australian Journal of Rural Health. 2021;29(3):464-72.

4. Lasimbang HB, Shoesmith W, Mohd Daud MNB, Kaur N, Jin MCP, Singh J, et al. Private troubles to public issue: empowering communities to reduce alcohol-related harm in Sabah, Malaysia. Health promotion international. 2017;32(1):122-9.

5. Geraldine A. Sabah govt respects right of non-Muslims to consume alcohol. New Straits Time. 2020.

6. Annuar SNS, Dawayan CR. Kaamatan Goes Virtual: Utilizing Social Media in Promoting Tourism Event. Digital Transformation and Innovation in Tourism Events: Routledge; 2022. p. 60-71.

7. Gimbad E. Cultivating Rice and Identity: An Ethnography of the Dusun People in Sabah, Malaysia [Master’s Thesis]: Western Sydney Universitiy; 2020.

8. Joseph A, Lasimbang HB, James S, Seok CB. Effects of alcohol towards quality of life in the indigenous groups of the West Coast Division, Sabah, Malaysia. Borneo Journal of Medical Sciences (BJMS). 2020;14(2):19-.

9. Tangit TM. Ethnic Labels and Identity among Kadazans in Penampang, Sabah (Malaysian Borneo) [Doctoral Dissertation]: The Australian National University; 2017.

10. Pang N, Lee G, Tseu M, Joss JI, Honey HA, Shoesmith W, et al. Validation of the alcohol use disorders identification test (AUDIT)–Dusun version in alcohol users in Sabahan Borneo. Archives of Psychiatry Research: An International Journal of Psychiatry and Related Sciences. 2020;56(2):129-42.

11. Mutalip MHBA, Kamarudin RB, Manickam M, Abd Hamid HAB, Saari RB. Alcohol consumption and risky drinking patterns in Malaysia: findings from NHMS 2011. Alcohol and alcoholism. 2014;49(5):593-9.

12. Joseph A. The Effect of Drinking Style on Alcohol-related Harm and Readiness to Change among the Indigenous Communities of Sabah. 2020.

13. Omar R, Ilmiyah C, Robinson F, a karim H. Qualitative Study of Barriers in Adopting a Healthy Diet and Lifestyle Among Kadazandusun Community with Nasopharyngeal Carcinoma in Sabah. International Journal of Scientific and Research Publications. 2022;12:507-13.

14. Shoesmith WD, Oo Tha N, Naing KS, Abbas RBH, Abdullah AF. Unrecorded alcohol and alcohol-related harm in rural Sabah, Malaysia: A socio-economically deprived region with expensive beer and cheap local spirits. Alcohol and Alcoholism. 2016;51(6):741-6.

15. Manickam MA, Abdul Mutalip MHB, Hamid HABA, Bt Kamaruddin R, Sabtu MYB. Prevalence, comorbidities, and cofactors associated with alcohol consumption among school-going adolescents in Malaysia. Asia Pacific Journal of Public Health. 2014;26(5_suppl):91S-9S.

16. Mutalip MHBA, Naidu BBM, Kamaruddin RB, Ab Hamid HAB, Ali NB, Ahmad NAB, et al. How Severe is Binge Drinking in Malaysia and Who are at Risk? Journal of Alcoholism & Drug Dependence. 2013;1(6):1-7.

17. Pérez RL. Fiesta as tradition, fiesta as change: ritual, alcohol and violence in a Mexican community. Addiction. 2000;95(3):365-73.

18. Corbin WR, Farmer NM, Nolen-Hoekesma S. Relations among stress, coping strategies, coping motives, alcohol consumption and related problems: A mediated moderation model. Addictive Behaviors. 2013;38(4):1912-9.

19. Hasking P, Lyvers M, Carlopio C. The relationship between coping strategies, alcohol expectancies, drinking motives and drinking behaviour. Addictive behaviors. 2011;36(5):479-87.

20. Kilian C, O'Donnell A, Potapova N, López‐Pelayo H, Schulte B, Miquel L, et al. Changes in alcohol use during the COVID‐19 pandemic in Europe: A meta‐analysis of observational studies. Drug and alcohol review. 2022;41(4):918-31.

21. Pollard MS, Tucker JS, Green HD. Changes in adult alcohol use and consequences during the COVID-19 pandemic in the US. JAMA network open. 2020;3(9):e2022942-e.

22. Bryden A, Roberts B, Petticrew M, McKee M. A systematic review of the influence of community level social factors on alcohol use. Health & place. 2013;21:70-85.

23. Nordfjærn T, Brunborg GS. Associations between human values and alcohol consumption among Norwegians in the second half of life. Substance use & misuse. 2015;50(10):1284-93.

24. Jernigan D, Noel J, Landon J, Thornton N, Lobstein T. Alcohol marketing and youth alcohol consumption: a systematic review of longitudinal studies published since 2008. Addiction. 2017;112:7-20.

25. Ryan SM, Jorm AF, Lubman DI. Parenting factors associated with reduced adolescent alcohol use: a systematic review of longitudinal studies. Australian & New Zealand Journal of Psychiatry. 2010;44(9):774-83.

26. Brisibe S, Ordinioha B, Dienye PO. Intersection between alcohol abuse and intimate partner's violence in a rural Ijaw community in Bayelsa State, South-South Nigeria. Journal of interpersonal violence. 2012;27(3):513-22.

27. Kaur R, Garg S. Domestic violence against women: A qualitative study in a rural community. Asia Pacific Journal of Public Health. 2010;22(2):242-51.

Gendered narratives and cultural shifts: A qualitative study on decadal changes in community alcohol consumption

Ming Gui Tan[1], Walton Wider[2], Nicholas Tze Ping Pang[3], Helen Benedict Lasimbang[3], Wendy Diana Shoesmith[3,4], Leilei Jiang[5], & Natchana Bhutasang[6,*]

[1]Department of Psychiatry and Psychological Health, Hospital Universiti Malaysia Sabah, Jalan UMS 88400 Kota Kinabalu Malaysia
[2]Faculty of Business and Communications, INTI International University, Nilai 71800, Negeri Sembilan, Malaysia
[3]Faculty of Medicine and Health Sciences, Universiti Malaysia Sabah, Jalan UMS 88400 Kota Kinabalu Malaysia
[4]Derbyshire Healthcare NHS Foundation Trust, Trust Headquarters Kingsway Hospital Derby Derbyshire DE22 3LZ
[5]Faculty of Education and Liberal Arts, INTI International University, Nilai 71800, Negeri Sembilan, Malaysia
[6]Faculty of Health Sciences, Shinawatra University, Pathum Thani 12160, Thailand
*Corresponding Author: natchana.b@mru.ac.th

Abstract

This study explores the impact of gender and cultural transformations on alcohol consumption within Sabah, Malaysia, over the last decade. Employing a qualitative approach, we conducted focus group interviews with members of the native community to delve into its evolving relationship with alcohol. The study reveals a significant shift in drinking habits. Historically, alcohol was mostly consumed by men, but now more women are drinking, changing long-standing gender roles related to alcohol use. Children often copy their parents' drinking behaviors, and with alcohol being more easily available, more people are drinking than before. Our findings also expose the complex consequences of alcohol use, which extend to health concerns, familial tension, and economic hardship. Despite the entrenched cultural status of alcohol, these negative outcomes are exacerbated by a lack of supportive healthcare services. In light of these insights, the study suggests the need for intervention plans that respect the cultural background of Sabah while tackling the current issues of alcohol misuse. The research adds to the wider conversation about managing alcohol in different cultural settings and highlights the importance of creating informed policies and health programs that are tailored to the specific needs of the community.

Keywords: Sabah community, Gendered alcohol perceptions, Alcohol-related interventions

Introduction

Sabah, a state that exhibits notable cultural and economic differences compared to West Malaysia, is recognized as one of the leading regions in the country with a significantly elevated prevalence of alcohol consumption (1). Sabah, situated on the island of Borneo, showcases distinctive socio-cultural dynamics notably shaped by the Kadazandusun community (2). Within this context, it is worth noting the significant prevalence of alcohol misuse (3, 4). In Sabah's traditional celebrations, alcohol consumption has been deeply ingrained throughout

history (5). This is especially true during the harvest festival known as the Kaamatan festival. This annual harvest ceremony holds great significance among the Kadazandusun community (6). In this context, it is observed that the consumption of traditional home-brewed alcoholic beverages such as Tapai and Montoku holds significance, as they have been historically associated with various ceremonial occasions including the New Year, Christmas, birthdays, and routine social gatherings (7-9).

Nevertheless, the influence of alcohol surpasses mere ceremonial practices. Alcohol has been found to have significant problematic implications in various aspects of Sabah, including domestic violence, absenteeism from work, motor accidents, and physical confrontations (4, 10). Approximately 23.6% of alcohol consumption in Sabah can be classified as "risky," indicating the significant nature of this matter (11). Paradoxically, the complex interplay of cultural elements presents a significant challenge in addressing the issue of alcohol misuse (12). The inherent conflict between traditional values and the consequences of intoxication creates a reluctance among individuals to openly acknowledge and confront their alcohol-related problems (10).

The consumption of alcohol is closely associated with cultural practices, particularly within indigenous communities (1). However, there is a lack of clear distinction between traditional usage and excessive consumption. For example, the Kadazan-Dusun community is acknowledged for its distinctive patterns of alcohol consumption and ceremonies involving alcoholic beverages (13). However, they also place significant emphasis on the principles of moderation, societal engagement, and spiritual obligations (2). The presence of contrasting elements within the cultural perception of alcohol underscores the need for a comprehensive and nuanced comprehension.

Our study explores how alcohol consumption in Sabah has changed due to shifts in gender roles and culture over the past decade. Initially, we provide a literature review to give background on the role of alcohol in the history and culture of Sabah. We then detail our methods, focusing on how we used focus group discussions and thematic analysis to understand alcohol use. The findings section reveals the influence of gender and culture on how people in Sabah view and use alcohol. This research adds to the ongoing conversation about alcohol consumption in the region. Our goal is to offer valuable insights and lay down a strong foundation for future work to create well-informed interventions.

Literature Review

Sociocultural, economic, and demographic factors are closely linked to the patterns of alcohol consumption in Sabah, Malaysia. In order to enhance our comprehension of these patterns, it is crucial to provide further details on the particular studies mentioned and their impacts on the field. Joseph et al. (8) highlighted the contrasting effects of alcohol by examining its influence on various aspects such as health, behavior, social dynamics, economy, and psychology. This study was utilized for its extensive analysis of the impacts of alcohol, which serves as a basis for comprehending the complex aspects of alcohol consumption in Sabah. The authors demonstrated that moderate alcohol consumption can yield beneficial effects on both individuals and communities. Nevertheless, the act of consuming an excessive amount of alcohol in an irregular manner results in substantial adverse outcomes, affecting not only the individuals who consume the alcohol but also their families and the broader

community. This differentiation emphasizes the subtle and sophisticated approach required when contemplating the role of alcohol in society.

Alcohol plays a crucial role in the cultural and traditional practices of the Indigenous communities in Sabah, serving as an essential component deeply woven into their social structure. In their study, Abd Rashid et al. (3) specifically examined the indigenous communities of Sabah. They discovered a troubling increase in alcohol consumption, which was influenced by gender norms and religious beliefs. This study is crucial as it emphasizes the distinctive elements specific to Sabah, setting it apart from the wider

Malaysian contexts and emphasizing the need for focused research in this region. Shoesmith et al. (14) conducted a comprehensive analysis of the various economic factors related to alcohol consumption, with a specific focus on the effects of taxation and smuggling. This research is crucial for comprehending the economic dynamics that impact alcohol consumption patterns in Sabah, providing localized solutions to tackle these challenges. In their analysis of alcohol consumption across Malaysia, Robert Lourdes et al. (1) and Mutalip et al. (11) broadened the scope to encompass demographic determinants, such as gender, age, education, employment, and smoking habits. These studies play a crucial role in identifying larger patterns and vulnerabilities, but their relevance to Sabah specifically requires careful examination of the local sociocultural context to ensure applicability. In addition, Manickam et al. (15) provided information regarding the frequency of alcohol consumption among Chinese students and Borneo natives, offering insights into the cultural foundations of alcohol consumption during ceremonies and social events. This study, in addition to the research conducted by Mutalip et al. (16) on the prevalence of binge drinking among the Bumiputera Sabah and Sarawak populations, highlights the importance of cultural factors in East Malaysia.

All together, these studies demonstrate the variety of factors influencing alcohol consumption patterns in Sabah and Malaysia at large. Nevertheless, the research deficiency exists in merging these discoveries to construct a thorough comprehension that is both culturally attuned and tailored to the distinct sociocultural, economic, and demographic conditions of Sabah. This study seeks to address this gap by integrating these observations into a coherent examination, pinpointing areas of consensus and discord in existing research, and explaining implications for policy and intervention approaches specifically designed for the Sabah context.

Methodology

Participants

The western coast is the focal point of the alcohol abuse epidemic due to cultural dynamics. Four zones in this region, namely Kota Kinabalu (KK), Kudat, Penampang, and Kiulu, became our target study areas, covering a vast geographical spread of 160km, thereby offering a diverse range of perspectives. The recruitment period started from 1 July 2020 to 31 August 2021.

One or more focus groups were formed in each target study area. A volunteer sampling method was utilised to recruit a group consisting of 6 to 7 study participants in each area. The youngest participant was 14 years old whereas the oldest was 81 years old. To incentivize participation, reimbursements were offered. Our study involved 7 focus group discussions, comprising 6 to 7 members each.

Procedure

Each discussion group was supervised by a research associate accompanied by a facilitator. Participants consisted of mixed genders. Group discussions were held in common gathering area of the community, such as a community hall. The facilitator's responsibilities included recruiting participants, recording the session, and transcribing the audio files. Upon beginning the session, the study's objectives and issues regarding confidentiality were clarified to the attendees. Subsequently, they were requested to provide their signatures on informed consent forms.

Interviews were primarily conducted in Bahasa Malaysia, the nation's official language. However, the local dialect was occasionally employed to facilitate a deeper comprehension. The interview of each focus group took about 1.5 hour and was based on the following prompt questions:

1. How has alcohol consumption in your community evolved over the past decade?
2. How do gender and culture affect the alcohol consumption in your community?

Once the interviews concluded, participants were granted reimbursements in diverse forms such as food supplies, T-shirts, and masks.

Reflexivity

The urban-centric perspective may limit researchers' insight into the cultural significance and accessibility of alcohol in the community. Additionally, the main researcher's psychiatric background introduces potential negative presumptions towards alcohol use, requiring careful consideration.

To address these challenges, researchers maintained reflective diaries to document and manage potential biases. Regular discussions with supervisors provided external perspectives, ensuring a more nuanced interpretation of the data. This reflexivity process aims to enhance the study's credibility by minimizing the impact of inherent biases on the qualitative analysis.

Ethical Considerations

During our qualitative research, we recognized participants might be hesitant to share due to concerns about community backlash and the worry of painting their community in a bad light. To address these concerns and ensure the accuracy of our data, we implemented strict measures to protect participants' identities. Instead of using names, we used alphabetical codes (A to G) and group numbers (1 to 7). Specific details about locations or other identifying information were intentionally left out. By taking these measures, we aimed to build trust and ensure participants felt comfortable sharing their genuine experiences.

Data Analysis

Initially, audio recordings from participant interviews were accurately transcribed verbatim. A third-party assistant with specialized training handled this transcription, aiming for both precision and an impartial reflection of participants' statements to minimize potential biases at the outset. Upon acquiring the transcribed data, our analysis proceeded through

manual thematic analysis. ChatGPT was employed to streamline the organization and management of data, ensuring efficient retrieval and categorization to support the research team's manual analysis.

Our analytical process began with a meticulous examination of the transcripts to uncover primary patterns or themes related to alcohol consumption and its community impact. The thematic analysis approach was systematic, incorporating several essential phases to guarantee its thoroughness and reliability.

During the code development phase, we generated a series of codes to categorize observations from the data. These codes emerged from recurring patterns and themes in the transcripts, particularly focusing on various aspects of alcohol consumption and its socio-cultural, economic, and demographic ramifications. Through iterative discussions within the research team, these initial codes were refined. This collaborative effort not only facilitated the identification of main themes but also ensured our analysis was grounded in the data and reflected the complexity of participants' experiences.

An expert review process was integral to evaluating the themes arising from our analysis. It involved soliciting feedback from seasoned researchers in the field and representatives of the studied community. By presenting our findings to these stakeholders during meetings, we could thoroughly vet our thematic conclusions, enhancing the credibility of our analysis. Furthermore, peer debriefing sessions served as an additional layer of scrutiny, providing vital oversight of the analytical process. These sessions enabled the research team to scrutinize and refine thematic conclusions, reinforcing the reliability and credibility of our findings.

Results

Based on all the responses gathered, four themes emerged.

The Cultural Backbone and Its Evolution

The consumption of alcohol in our community is deeply intertwined with traditions and changing perceptions. Historically, alcohol has played a pivotal role in celebrations and rites of passage, a sentiment echoed by a respondent who remarked on the inescapability of tradition, "...cannot escape tradition..." (B6). Another respondent highlighted the ceremonial importance of sharing the first alcoholic drink with parents, signifying a rite of passage that was once deeply respected. However, contemporary narratives suggest a shift from these traditional moorings. One respondent captured this evolution, stating, "It feels like the traditional meaning has been lost now. People used to drink to celebrate, but now it's more to run away from problems." (F1). This indicates a profound change in the role and significance of alcohol within the community—from a symbol of joy and communal bonding to a means of escape from personal and societal issues.

The societal upheavals brought on by the COVID-19 pandemic have further influenced alcohol consumption habits. A stark portrayal of this change was shared by a participant: "The use of alcohol has changed from the past because there was no COVID-19 pandemic before; people spent time going to work to earn a living. Now, because of the COVID-19 pandemic, many are out of work, many don't know what to do, and they drink alcohol to kill time." (F1) This reflection not only underscores the impact of the pandemic on daily life and employment

but also highlights the adaptive—or maladaptive—shifts in alcohol consumption as a coping mechanism. Amidst this evolving environment, researchers observed the emergence of distinct patterns of consumption, characterized by some individuals attempting to moderate their consumption while others engaging in excessive indulgence. Particularly concerning is the trend of underage drinking, a deviation from traditional norms potentially fuelled by generational influences. "However, a few of them who are not of legal drinking age have indeed been taking it secretly without their parents' knowledge." (G6), reveals the normalization of alcohol consumption among the youth, challenging the community's established norms and expectations.

The Double-Edged Sword of Visibility and Accessibility

The alcohol availability in our community has experienced substantial changes, as highlighted by a respondent's observation: "In this place, there are no obstacles or prohibitions to buying alcohol" (E2), illustrating the ease with which alcohol can now be purchased. This lack of restrictions has opened the door to various means of access, including the tradition of homebrewing. One participant shared their experience with localized production: "Here, we used to make our own alcohol using rice and medicine, and it had to be made for a week before it could be consumed" (A3), highlighting the ability in making homemade alcohol with everyday ingredients.

The journey from scarcity to abundance is further articulated by another respondent, who notes the stark contrast between past and present: "It used to be hard to find stores that sold alcohol. Now they are almost on every corner" (A2). This change points to a dramatic increase in the physical visibility of alcohol, making it a commonplace feature in the community's landscape. Echoing this sentiment, another adds, "Alcohol is cheaper and easier to find compared to ten years ago" (F7), indicating not only increased availability but also affordability over time. The ubiquity of alcohol is captured in the routine experiences of community members, as one person remarked, "...we often go into Speedmart 99. When we go in, we see it there." (E6). This statement reflects how alcohol has become a regular item on the shopping list, seamlessly integrated into daily life and consumer habits.

Navigating Social Influences and Evolving Gender Norms in Alcohol Consumption

The patterns of alcohol consumption in our community are greatly influenced by peer pressure and changing gender norms, which demonstrate the interaction of social constructs. One respondent encapsulated the peer pressure phenomenon among the youth, stating, "Sometimes, when consumed among teenagers, it is referred to as 'psycho', wanting to show oneself as someone strong" (G1). This insight reveals how, in some circles, alcohol consumption among teenagers is tied to displays of strength or bravado. The social aspect of drinking is further emphasized by another participant: "Moreover, if there are friends, they meet up and drink straight away. I'm the same, if initially I drink a little, over time it becomes until I'm drunk, and only after I've had enough do I sleep" (D3). Such statements underline the influence of peer gatherings on alcohol consumption patterns, suggesting a trend where socializing often escalates to excessive drinking.

A particularly noteworthy shift is observed in the gender dynamics of alcohol consumption. Historically dominated by men, the practice has seen increasing participation

from women. "I remember before only men drank. Now, I see many women also drinking" (A4), one respondent remarked, highlighting this changing trend. Furthermore, women's involvement in alcohol production, traditionally a male-dominated activity, signifies a notable shift towards gender equality in this realm: "Before, I thought only men could make alcohol at home. Now, I see many women also trying to make it themselves" (C4). Despite these advancements, societal judgments persist, with a discernible bias in how male and female drinkers are perceived: "There's a difference in our society. If a man drinks, people say he's strong. But if a woman drinks, they look down on her" (B4). This dichotomy suggests that while progress has been made, deep-seated cultural norms and expectations still influence the acceptance of female alcohol consumption.

Family influence also plays a pivotal role in shaping attitudes and behaviors toward alcohol. The narrative, "If parents drink, it's highly likely their children will also drink" (A2), points to the generational transmission of drinking habits, underscoring the impact of parental behavior on children. This notion of familial legacy in alcohol consumption is further supported by another participant, who observed, "...it's been from long ago, from parents, grandparents, they indeed drank. So we, our generation now, follow in their footsteps" (E3). Such observations highlight the significance of family in the continuum of alcohol consumption practices within the community.

The Multifaceted Impact of Alcohol on Life's Dimensions

The consumption of alcohol casts a wide net of repercussions, affecting various aspects of life such as health, relationships, work, and financial stability. The journey into alcohol use often begins with experimentation in youth, leading some to discover its counterproductive effects on personal problems. As one individual poignantly reflects, "I used to drink to forget my problems. But now I realize it only adds to the problems" (D2), this realization underscores the ineffectiveness of alcohol as a coping mechanism.

Health concerns associated with alcohol consumption are a significant worry for many, with individuals noting the detrimental effects on their well-being. "I try to stop drinking because I feel it affects my health" (D2), reveals the conscious decision to mitigate these health risks by reducing alcohol intake. The acknowledgment of alcohol's role in exacerbating health issues highlights the need for awareness and intervention in managing consumption. Beyond personal health, alcohol's ripple effects extend to the fabric of familial relationships, with some observing its capacity to strain bonds within the family unit. "I feel drinking alcohol can damage health and family relationships" (D7), encapsulates the broader social consequences of alcohol use, suggesting that its impact reaches beyond the individual to touch the lives of those around them. The workplace is not immune to the influences of alcohol, as evidenced by accounts of job losses attributed to drinking. "I know several people who lost their jobs because they got drunk at work" (F1), points to the severe implications of alcohol misuse in a professional setting, affecting not only individual careers but also organizational productivity and safety. Financial strain is another critical concern tied to alcohol consumption. The shift from spending on alcohol to prioritizing family finances, as shared by one respondent, "I used to spend a lot of money on alcohol. Now I try to save that money for my family" (D7), reflects a growing awareness of the economic toll of excessive drinking. This perspective shift underscores the recognition of the need to reallocate resources more responsibly.

Overall, the stories surrounding alcohol consumption demonstrate an interaction of negative consequences in terms of health, relationships, employment, and finances. These testimonies emphasize the difficulties presented by alcohol, prompting a more thorough analysis of its impact on society and individuals' lives. To tackle these concerns, a comprehensive strategy is needed that combines health education, support networks, and community involvement to navigate the complex effects of alcohol on various aspects of life.

Discussion

The discussion reveals a nuanced understanding of the evolving patterns of alcohol consumption within the Sabah community, particularly along its western coast, elucidating the intricate interplay of cultural, social, and health-related factors. Historically, alcohol has been deeply ingrained in the community's cultural fabric, playing a central role in ceremonies and celebrations, akin to many indigenous societies globally (17). Traditional alcoholic beverages like tapai, lihing, tumpung, and bahar have symbolized communal unity and ancestral customs, though their significance has evolved over time, with alcohol now serving as a coping mechanism for some individuals amidst life's challenges (18, 19).

The dynamics of alcohol consumption have undergone shifts, influenced by significant events such as the COVID-19 pandemic and the subsequent Movement Control Order (MCO) in Malaysia. While urban areas experienced a reduction in alcohol consumption due to restrictions on mass gatherings, rural regions witnessed a continuation of traditional drinking habits, indicating a geographical shift in consumption patterns (20). These changes reflect broader global trends where societal stressors drive individuals towards substance use (20, 21). Additionally, community-level availability and marketing of alcohol play a role in shaping consumption behaviors, particularly among adolescents (22).

The discussion also highlights the influence of age, urbanization, and beverage preferences on alcohol consumption. Older generations in rural areas tend to favor traditional homemade beverages, whereas younger demographics in urban settings lean towards commercially available alcoholic drinks (23). Youth involvement in alcohol consumption is concerning, with peer influence and societal pressures driving premature and potentially hazardous drinking habits (24). Furthermore, the evolving gender dynamics in alcohol consumption reveal a transition towards increased participation of women, reflecting broader shifts in gender roles, albeit with lingering gendered perceptions and societal judgments (3). Family dynamics and community regulations also shape alcohol-related behaviors, with parental attitudes towards alcohol influencing offspring in complex ways (25).

The detrimental effects of alcohol on health, relationships, and domestic violence underscore the need for comprehensive interventions, including educational counselling services on moderate alcohol use (26, 27). Despite the inadequacy of health services related to alcohol, initiatives driven by religious affiliations have dominated legislative discussions, indicating a gap in addressing core issues (27). Overall, the discussion emphasizes the multifaceted nature of alcohol consumption within the Sabah community and the need for tailored interventions to address its cultural, social, and health-related dimensions.

Limitations and Recommendations for Future Research

The research presented primarily focuses on the western coast of the Sabah community, which might not capture the entirety of practices and perspectives prevalent in other regions within Sabah. This geographical focus presents a limitation in understanding the full depth and breadth of alcohol consumption patterns within the broader Sabah community. Moreover, the data captured in the study is up to the present time, and given the fluid nature of societal perspectives and practices, the findings might not encapsulate potential future shifts.

Another significant consideration is the background of the researcher as a healthcare staff member. This may have introduced a potential bias in the way participants responded, as they might either consciously or subconsciously provide answers they deemed 'acceptable' or 'expected' within a healthcare context. This could particularly influence responses related to health outcomes, services, and perceptions of alcohol's effects.

Additionally, while the study emphasizes the historical and cultural importance of alcohol to the community, there might be limitations in the depth of exploration concerning specific rites, rituals, and traditions surrounding its use. Addressing the sensitive aspects of the research, especially around gender dynamics and the role of alcohol in domestic issues, might also have been influenced by reporting biases, especially in areas like domestic violence.

For a more comprehensive understanding of the relationship of the Sabah community with alcohol, it would be beneficial to expand the geographical scope of future studies. This would ensure a richer understanding by capturing diverse nuances within the entire community. A longitudinal approach, observing and noting changes over extended periods, could provide invaluable insights into the evolving patterns of alcohol consumption and its broader implications.

Given the potential bias introduced due to the researcher's background, future studies should consider involving a diverse team of researchers from various backgrounds, ensuring a more neutral data collection process. This could alleviate participants' concerns and encourage more candid responses.

A dedicated focus on the specific cultural and ceremonial uses of alcohol might offer a deeper dive into its traditional significance. Collaborating across disciplines with anthropologists, sociologists, and health professionals can paint a more holistic picture.

Alarming trends among the youth have been highlighted in the findings, pointing to a need for more in-depth studies targeting this demographic. Feedback on the inadequacy of health services related to alcohol since 2017 also indicates a gap that needs addressing. Evaluating these services in detail could highlight areas needing improvement.

Lastly, considering the effectiveness of localized strategies, like village customary laws, could pave the way for designing interventions that resonate with the community. Future research can thus address current limitations and provide a robust foundation to tackle the multifaceted challenges faced by the Sabah community in relation to alcohol.

Conclusion

The narratives from the Sabah community reveal a complex interplay in the dynamics of alcohol consumption. While its cultural significance remains undeniable, changing perceptions, evolving gender roles, and global events like the pandemic underscore the seismic

shift in the community's relationship with alcohol. As global influences intersect with local traditions, the emerging challenges are multifaceted and demand an intricate understanding of the evolving patterns and their foundational societal structures. Addressing these transitions necessitates a holistic approach that both honors the deeply embedded cultural roots and confronts the contemporary challenges. It is imperative to champion community-based solutions, elevate awareness, and untangle the intricate web of influences to effectively navigate this shifting landscape.

References

1. Robert Lourdes TG, Abd Hamid HA, Riyadzi MR, Rodzlan Hasani WS, Abdul Mutalip MH, Abdul Jabbar N, et al. Findings from a nationwide study on alcohol consumption patterns in an upper middle-income country. International Journal of Environmental Research and Public Health. 2022;19(14):8851.

2. Wider W, Suki NM, Lott ML, Nelson LJ, Low SK, Cosmas G. Examining criteria for adulthood among young people in sabah (East Malaysia). Journal of Adult Development. 2021;28(3):194-206.

3. Abd Rashid RB, Mohd Daud MNB, Guad RM, Gan SH, Wan Husin WAIB, Giloi N, et al. Prevalence and risk factors associated with alcohol consumption among indigenous people in Sabah Borneo Island. Australian Journal of Rural Health. 2021;29(3):464-72.

4. Lasimbang HB, Shoesmith W, Mohd Daud MNB, Kaur N, Jin MCP, Singh J, et al. Private troubles to public issue: empowering communities to reduce alcohol-related harm in Sabah, Malaysia. Health promotion international. 2017;32(1):122-9.

5. Geraldine A. Sabah govt respects right of non-Muslims to consume alcohol. New Straits Time. 2020.

6. Annuar SNS, Dawayan CR. Kaamatan Goes Virtual: Utilizing Social Media in Promoting Tourism Event. Digital Transformation and Innovation in Tourism Events: Routledge; 2022. p. 60-71.

7. Gimbad E. Cultivating Rice and Identity: An Ethnography of the Dusun People in Sabah, Malaysia [Master’s Thesis]: Western Sydney Universitiy; 2020.

8. Joseph A, Lasimbang HB, James S, Seok CB. Effects of alcohol towards quality of life in the indigenous groups of the West Coast Division, Sabah, Malaysia. Borneo Journal of Medical Sciences (BJMS). 2020;14(2):19-.

9. Tangit TM. Ethnic Labels and Identity among Kadazans in Penampang, Sabah (Malaysian Borneo) [Doctoral Dissertation]: The Australian National University; 2017.

10. Pang N, Lee G, Tseu M, Joss JI, Honey HA, Shoesmith W, et al. Validation of the alcohol use disorders identification test (AUDIT)–Dusun version in alcohol users in Sabahan Borneo. Archives of Psychiatry Research: An International Journal of Psychiatry and Related Sciences. 2020;56(2):129-42.

11. Mutalip MHBA, Kamarudin RB, Manickam M, Abd Hamid HAB, Saari RB. Alcohol consumption and risky drinking patterns in Malaysia: findings from NHMS 2011. Alcohol and alcoholism. 2014;49(5):593-9.

12. Joseph A. The Effect of Drinking Style on Alcohol-related Harm and Readiness to Change among the Indigenous Communities of Sabah. 2020.

13. Omar R, Ilmiyah C, Robinson F, a karim H. Qualitative Study of Barriers in Adopting a Healthy Diet and Lifestyle Among Kadazandusun Community with Nasopharyngeal Carcinoma in Sabah. International Journal of Scientific and Research Publications. 2022;12:507-13.

14. Shoesmith WD, Oo Tha N, Naing KS, Abbas RBH, Abdullah AF. Unrecorded alcohol and alcohol-related harm in rural Sabah, Malaysia: A socio-economically deprived region with expensive beer and cheap local spirits. Alcohol and Alcoholism. 2016;51(6):741-6.

15. Manickam MA, Abdul Mutalip MHB, Hamid HABA, Bt Kamaruddin R, Sabtu MYB. Prevalence, comorbidities, and cofactors associated with alcohol consumption among school-going adolescents in Malaysia. Asia Pacific Journal of Public Health. 2014;26(5_suppl):91S-9S.

16. Mutalip MHBA, Naidu BBM, Kamaruddin RB, Ab Hamid HAB, Ali NB, Ahmad NAB, et al. How Severe is Binge Drinking in Malaysia and Who are at Risk? Journal of Alcoholism & Drug Dependence. 2013;1(6):1-7.

17. Pérez RL. Fiesta as tradition, fiesta as change: ritual, alcohol and violence in a Mexican community. Addiction. 2000;95(3):365-73.

18. Corbin WR, Farmer NM, Nolen-Hoekesma S. Relations among stress, coping strategies, coping motives, alcohol consumption and related problems: A mediated moderation model. Addictive Behaviors. 2013;38(4):1912-9.

19. Hasking P, Lyvers M, Carlopio C. The relationship between coping strategies, alcohol expectancies, drinking motives and drinking behaviour. Addictive behaviors. 2011;36(5):479-87.

20. Kilian C, O'Donnell A, Potapova N, López‐Pelayo H, Schulte B, Miquel L, et al. Changes in alcohol use during the COVID‐19 pandemic in Europe: A meta‐analysis of observational studies. Drug and alcohol review. 2022;41(4):918-31.

21. Pollard MS, Tucker JS, Green HD. Changes in adult alcohol use and consequences during the COVID-19 pandemic in the US. JAMA network open. 2020;3(9):e2022942-e.

22. Bryden A, Roberts B, Petticrew M, McKee M. A systematic review of the influence of community level social factors on alcohol use. Health & place. 2013;21:70-85.

23. Nordfjærn T, Brunborg GS. Associations between human values and alcohol consumption among Norwegians in the second half of life. Substance use & misuse. 2015;50(10):1284-93.

24. Jernigan D, Noel J, Landon J, Thornton N, Lobstein T. Alcohol marketing and youth alcohol consumption: a systematic review of longitudinal studies published since 2008. Addiction. 2017;112:7-20.

25. Ryan SM, Jorm AF, Lubman DI. Parenting factors associated with reduced adolescent alcohol use: a systematic review of longitudinal studies. Australian & New Zealand Journal of Psychiatry. 2010;44(9):774-83.

26. Brisibe S, Ordinioha B, Dienye PO. Intersection between alcohol abuse and intimate partner’s violence in a rural Ijaw community in Bayelsa State, South-South Nigeria. Journal of interpersonal violence. 2012;27(3):513-22.

27. Kaur R, Garg S. Domestic violence against women: A qualitative study in a rural community. Asia Pacific Journal of Public Health. 2010;22(2):242-51.

Response to Reviewers

Dear Reviewers,

Thank you for your review. We have made necessary changes and improvements based on your review comments, which are as below. We have also added 2 additional co-authors who have diligently helped in the revision of the manuscript.

Reviewer 1

Comments	Remarks
My only comment is that you review the form of citation of the document, since they must be surnames and in some citations they have names, just verify according to the editorial guidelines. It is a good article and of great importance for theoretical bases of this problem.	Thank you for the comments. We have revised accordingly.

Reviewer 2

Comments	Remarks
This manuscript on gendered narratives and cultural shifts in alcohol consumption in the Sabah community has some potential. However, I found it wasn't guided by a clear research question	We have made amendments and came up with a stronger research question.
I'm also not sure this is the most appropriate journal for it (perhaps a journal more oriented sociological/cultural studies and substance use)	We feel that PLOS One is a suitable journal, as alcohol use is a very interdimensional and intertextual disorder that cuts across medicine, psychology, sociology and gender, hence we do not want to limit the scope by sending it to a very specific journal, but rather to a broad based journal.
I found the abstract difficult to follow – it jumped around without contextualising Sabah or providing a clear research question. More detail is needed here, and I would suggest a more structured abstract based off the introduction, methods, results and conclusion might be helpful.	We have made necessary amendments according to this comment.
The structure of the paper needs to be outlined much earlier in the introduction, which I think will help. While the information is there, the current structure doesn't always flow very coherently between topics and ideas	We have restructured the introduction to present the outline clearly.
You state the "study seeks to examine the trajectory of alcohol consumption within the Sabah community over the years" but I'm not sure you can do this with a study of only one timepoint. Perhaps it might be better to phrase this as reflections of cultural and gender shifts in alcohol consumption in Sabah	This has been amended.
The studies raised in the literature need elaboration – what did the studies do and why	We've revised the paper to provide more details on cited studies, clarified their distinctions, and

did you draw on them? Some of the studies seem to be focused on Sabah but others are drawn from Malaysia more broadly. What can/can't these studies tell us? Where is the research gap you are filling?	emphasized our contribution to filling the research gap
The methods are generally ok, but going through the COREQ checklist may help clarify some things. Importantly, the "Data Analysis" section is very underdone. How was the data "processed manually"? Was thematic analysis used? If so, how was it used? I'm also a little unsure about the use of ChatGPT as a tool to assist with analysis given the potential for bias/inaccuracies	We reviewed the COREQ checklist to ensure clarity in our methods. We acknowledged the need for further detail in the "Data Analysis" section and elaborated on how the data was processed manually through thematic analysis. Regarding the use of ChatGPT, we understood your concerns about bias and inaccuracies and provided additional justification for its inclusion or considered alternative methods.
I found many of the themes too brief to provide substantial analysis and interpretation. Lots of words were used up writing out both translations of quotes (authors should decide if this is worth the wordcount used) and I didn't feel like the themes told enough of a coherent story. I would consider collapsing themes if/where possible, but also using a reformulating the themes so they don't read like lists of quotes from participants	Thank you for your feedback. We have reviewed the themes and acknowledged the brevity you mentioned. We reconsidered the use of wordcount on translations of quotes and made adjustments. We collapsed themes where possible and reformulated them to ensure they provide a more coherent narrative, rather than reading like lists of participant quotes.
I felt the discussion spent a lot of time reviewing the literature, but didn't incorporate the findings to draw out comparisons/differences from previous research. A stronger discussion that draws on both relevant literature alongside your findings would provide clearer implications/conclusions for the paper.	We have revised the discussion so that it is now more concise now and draws out comparisons/differences from previous research.

Thank you.
