# Supplementary material for: Gendered narratives and cultural shifts: A qualitative study on decadal changes in community alcohol consumption
Source: PLoS One. 2024 Dec 2;19(12):e0309378. doi: 10.1371/journal.pone.0309378 (PMC11611190; doi:10.1371/journal.pone.0309378)
Supplement: Attachment — Submitted filename: Response to Reviewers.docx [file pone.0309378.s001.docx]

**Response to Reviewers**

Dear Reviewers,

Thank you for your review. We have made necessary changes and improvements based on your review comments, which are as below. We have also added 2 additional co-authors who have diligently helped in the revision of the manuscript.

Reviewer 1

| Comments | Remarks |
| --- | --- |
| My only comment is that you review the form of citation of the document, since they must be surnames and in some citations they have names, just verify according to the editorial guidelines. It is a good article and of great importance for theoretical bases of this problem. | Thank you for the comments. We have revised accordingly. |

Reviewer 2

| Comments | Remarks |
| --- | --- |
| This manuscript on gendered narratives and cultural shifts in alcohol consumption in the Sabah community has some potential. However, I found it wasn’t guided by a clear research question | We have made amendments and came up with a stronger research question. |
| I’m also not sure this is the most appropriate journal for it (perhaps a journal more oriented sociological/cultural studies and substance use) | We feel that PLOS One is a suitable journal, as alcohol use is a very interdimensional and intertextual disorder that cuts across medicine, psychology, sociology and gender, hence we do not want to limit the scope by sending it to a very specific journal, but rather to a broad based journal. |
| I found the abstract difficult to follow – it jumped around without contextualising Sabah or providing a clear research question. More detail is needed here, and I would suggest a more structured abstract based off the introduction, methods, results and conclusion might be helpful. | We have made necessary amendments according to this comment. |
| The structure of the paper needs to be outlined much earlier in the introduction, which I think will help. While the information is there, the current structure doesn’t always flow very coherently between topics and ideas | We have restructured the introduction to present the outline clearly. |
| You state the “study seeks to examine the trajectory of alcohol consumption within the Sabah community over the years” but I’m not sure you can do this with a study of only one timepoint. Perhaps it might be better to phrase this as reflections of cultural and gender shifts in alcohol consumption in Sabah | This has been amended. |
| The studies raised in the literature need elaboration – what did the studies do and why did you draw on them? Some of the studies seem to be focused on Sabah but others are drawn from Malaysia more broadly. What can/can’t these studies tell us? Where is the research gap you are filling? | We've revised the paper to provide more details on cited studies, clarified their distinctions, and emphasized our contribution to filling the research gap |
| The methods are generally ok, but going through the COREQ checklist may help clarify some things. Importantly, the “Data Analysis” section is very underdone. How was the data “processed manually”? Was thematic analysis used? If so, how was it used? I’m also a little unsure about the use of ChatGPT as a tool to assist with analysis given the potential for bias/inaccuracies | We reviewed the COREQ checklist to ensure clarity in our methods. We acknowledged the need for further detail in the "Data Analysis" section and elaborated on how the data was processed manually through thematic analysis. Regarding the use of ChatGPT, we understood your concerns about bias and inaccuracies and provided additional justification for its inclusion or considered alternative methods. |
| I found many of the themes too brief to provide substantial analysis and interpretation. Lots of words were used up writing out both translations of quotes (authors should decide if this is worth the wordcount used) and I didn’t feel like the themes told enough of a coherent story. I would consider collapsing themes if/where possible, but also using a reformulating the themes so they don’t read like lists of quotes from participants | Thank you for your feedback. We have reviewed the themes and acknowledged the brevity you mentioned. We reconsidered the use of wordcount on translations of quotes and made adjustments. We collapsed themes where possible and reformulated them to ensure they provide a more coherent narrative, rather than reading like lists of participant quotes. |
| I felt the discussion spent a lot of time reviewing the literature, but didn’t incorporate the findings to draw out comparisons/differences from previous research. A stronger discussion that draws on both relevant literature alongside your findings would provide clearer implications/conclusions for the paper. | We have revised the discussion so that it is now more concise now and draws out comparisons/differences from previous research. |

Thank you.
