# Supplementary material for: Gendered narratives and cultural shifts: A qualitative study on decadal changes in community alcohol consumption
Source: PLoS One. 2024 Dec 2;19(12):e0309378. doi: 10.1371/journal.pone.0309378 (PMC11611190; doi:10.1371/journal.pone.0309378)
Supplement: Attachment — Submitted filename: Reviewer Comment Checklist 2.docx [file pone.0309378.s003.docx]

**Reviewer’s Comments**

Reviewer 3

| Comments | Remarks |
| --- | --- |
| 1.In the abstract, start off with a brief introduction to the background of the topic and pain points first. This will then give a good low towards how the research question came about and what the research aims to achieve. The objectives has to be made clearer. | Amended as suggested. |
| 2.I would suggest to highlight the increased alcohol consumption among women in the introduction as well because exploring the shifts in gender role is one of the main objectives that your study is set out to achieve. | Amended as suggested. |
| 3. What is the conceptual framework used to guide this study? | The conceptual framework is described as suggested. |
| 4. How was the topic guide developed? | Described as suggested. |
| 5. Mention the type of reimbursement offered | Mentioned as suggested. |
| 6. Mention where did the study attain its ethical approval from. | Mentioned as suggested. |
| 7. Mention how was consent taken from those aged 18 years and below and was there any intervention suggested or given to minors who admitted to consuming alcohol. | Mentioned as suggested. |

Reviewer 4

| Comments | Remarks |
| --- | --- |
| Given the multifaceted nature of alcohol consumption in the Sabah community, I suggest the authors incorporate Bronfenbrenner's social-ecological model as a theoretical lens. This model can comprehensively capture the influences on behavior from individual to societal levels. By adopting this approach, the research can systematically analyze how factors like personal values, gender roles, family dynamics, societal perceptions, and community regulations intersect to shape alcohol consumption patterns. This comprehensive understanding will inform the development of community-centric solutions addressing multiple levels of influence, thus enhancing the effectiveness of interventions. | The Bronfenbrenner's social-ecological model was used as theoretical lens as suggested. We have also incorporated the social role theory. |
| Introduction:  The introduction provides a thorough overview of the cultural significance and prevalence of alcohol consumption in Sabah, particularly within the Kadazandusun community. However, one major weakness is the lack of clear delineation between the historical context of alcohol use in ceremonial practices and the contemporary issues of alcohol misuse. While the introduction acknowledges the problematic implications of alcohol consumption, it does not sufficiently connect these issues to the broader socio-cultural dynamics of Sabah. Additionally, the introduction could benefit from a clearer articulation of the research gap or question that the study aims to address. Establishing this gap would provide a clearer context for the significance of the research and guide the reader towards the study's objectives. | Research gap is clearer now. |
| Literature Review:  The literature review presents a broad overview of existing studies on alcohol consumption patterns in Sabah, Malaysia. However, it lacks critical engagement and synthesis of the literature, which diminishes its effectiveness. While summarizing the findings of various studies, the review fails to analyze the strengths and limitations of each study or to identify potential gaps or contradictions in the literature. A more critical approach would involve evaluating the methodological rigor of the studies, discussing the implications of their findings, and synthesizing the literature to highlight areas where further research is needed. Additionally, the review would benefit from a clearer articulation of how each study contributes to understanding alcohol consumption in Sabah specifically. I suggest the authors revise the literature review to provide a more in-depth analysis of existing research, critically evaluate the strengths and weaknesses of each study, and identify gaps in the literature that their study aims to address. This will enhance the overall quality and relevance of the literature review to the current study's objectives. | Revised as suggested. |
| Participants:  The major weakness of this section lies in the lack of detail regarding the selection criteria for participants and the rationale behind choosing the specific study areas. The section would benefit from providing more information on why these four zones were selected and how they represent the broader population of the western coast in terms of cultural dynamics and alcohol abuse. Additionally, while the age range of participants is mentioned, there is no explanation of how participants were recruited within this age range or whether any specific criteria were used to ensure diverse perspectives. To improve this section, the authors could provide a clearer rationale for the selection of study areas and participants, outline any specific inclusion criteria, and detail the recruitment process to ensure transparency and rigor in participant selection. Furthermore, including information on any efforts made to ensure diversity within the focus groups, such as considering gender, socioeconomic status, or ethnicity, would strengthen the overall methodology. | Revised as suggested. |
| Procedure:  The major weakness of this section lies in its brevity and lack of detail regarding the procedure followed during the focus group discussions. While it outlines the general setup and duration of the interviews, it lacks specific information on the facilitation process, the structure of the discussions, and any measures taken to ensure the validity and reliability of the data collected. Additionally, there is no mention of any efforts made to address potential biases or power dynamics within the focus groups, such as ensuring equal participation among all participants or mitigating social desirability bias. To improve this section, the authors could provide a more comprehensive description of the facilitation process, including how the facilitator encouraged open dialogue and ensured all voices were heard. They should also detail any strategies used to maintain confidentiality and obtain informed consent from participants. Furthermore, discussing how the interview questions were developed and piloted could enhance the transparency and credibility of the research methodology. Finally, including information on how the data were analyzed, such as through thematic analysis or qualitative coding, would provide insight into the rigor of the research process. | Revised as suggested. Data analysis will be discussed in another section, under “Data Analysis” |
| Reflexivity:  This section on reflexivity effectively acknowledges potential biases and challenges inherent in the research process, such as the urban-centric perspective and the researcher's background. It demonstrates a thoughtful approach to addressing these issues through the use of reflective diaries and regular discussions with supervisors. These measures aim to enhance the credibility of the study by minimizing the impact of biases on data interpretation. Overall, the section appears well-considered and does not necessarily require revision. However, if the authors wish to provide additional detail on how reflexivity was integrated throughout the research process or how specific biases were identified and addressed, they could consider expanding this section for greater clarity and transparency. | Refined as suggested. |
| Ethical consideration:  This section effectively addresses the ethical considerations related to participant confidentiality and the potential for community backlash. It outlines the measures taken to protect participants' identities, such as using alphabetical codes and omitting specific details about locations. By implementing these strict measures, the researchers aimed to build trust and create a safe environment for participants to share their experiences openly. Overall, the section appears well-written and does not necessarily require revision. However, if the authors wish to provide additional detail on how participant confidentiality was maintained throughout the research process or how participants were informed about these measures, they could consider expanding this section for further clarity. | Ethics approval committee was additionally mentioned. |
| Data analysis:  Strengths:  • Rigorous Transcription Process: The use of a third-party assistant with specialized training for accurate verbatim transcription minimizes potential biases and ensures the reliability of the data.  • Comprehensive Analytical Approach: The combination of manual thematic analysis and the utilization of ChatGPT for data organization demonstrates a thorough and systematic approach to data analysis.  • Iterative Code Development: The collaborative effort within the research team to develop and refine codes ensures that the analysis is grounded in the data and reflects the complexity of participants' experiences.  • Expert Review Process: Seeking feedback from seasoned researchers and community representatives enhances the credibility of the analysis and strengthens the validity of the findings.  Weaknesses:  • Lack of Detail on Analytical Techniques: While the section outlines the general analytical process, it could benefit from providing more explicit details on the specific techniques used during thematic analysis and how ChatGPT was employed to support the manual analysis.  • Limited Explanation of Peer Debriefing Sessions: Although mentioned, the section does not elaborate on how peer debriefing sessions were conducted or how they contributed to the analytical process.  Suggestions for Improvement:  • Provide More Detail on Analytical Techniques: The authors can enhance this section by explicitly outlining the steps involved in thematic analysis, including how themes were identified, coded, and refined. Additionally, they should explain in more detail how ChatGPT was utilized to support the manual analysis process.  • Elaborate on Peer Debriefing Sessions: The authors should provide a more detailed description of how peer debriefing sessions were conducted, including who participated, the frequency of sessions, and how feedback was incorporated into the analysis. This will strengthen the transparency and credibility of the analytical process. | Reviewed and revised as suggested. |
| Result  The Cultural Backbone and Its Evolution:  While the analysis under this theme adequately acknowledges the historical significance of alcohol and its evolving role within the community, it falls short in providing a deeper exploration of the underlying drivers behind the observed shifts in consumption patterns. The narrative touches upon the impact of external factors like the COVID-19 pandemic but lacks a comprehensive examination of the socio-economic, cultural, and psychological forces influencing these changes. Furthermore, there is a notable oversight in the analysis of underage drinking, with insufficient exploration of the factors contributing to its normalization and the potential repercussions for individuals and the community at large. Addressing these shortcomings would enhance the depth and criticality of the analysis, offering a more nuanced understanding of the complex dynamics surrounding alcohol consumption within the Sabah community. | Revised as suggested. |
| The Double-Edged Sword of Visibility and Accessibility:  The analysis of findings under this theme adequately portrays the shift in alcohol availability and accessibility within the community, highlighting the ease with which alcohol can now be obtained compared to previous years. However, there is a lack of critical examination regarding the implications of this increased accessibility on alcohol-related behaviors and societal norms. The analysis fails to delve into the potential consequences of widespread alcohol availability, such as increased rates of alcohol misuse, underage drinking, or alcohol-related harm. Furthermore, while the narrative touches upon the affordability of alcohol, there is limited exploration of how changes in pricing may impact consumption patterns and socioeconomic disparities within the community. By addressing these gaps, the analysis could offer a more comprehensive understanding of the complex dynamics surrounding alcohol accessibility and its implications for community well-being. |  |
| Navigating Social Influences and Evolving Gender Norms in Alcohol Consumption:  The analysis of findings under this theme effectively highlights the influence of social constructs, such as peer pressure and evolving gender norms, on alcohol consumption patterns within the community. However, there is a notable absence of deeper exploration into the underlying reasons for these shifts in social dynamics and their broader implications. For instance, while the narrative touches upon the increasing participation of women in alcohol consumption and production, there is limited discussion on the societal factors driving this change or the potential consequences for gender equality and social norms. Additionally, while family influence is acknowledged, the analysis could benefit from a more nuanced examination of how intergenerational transmission of drinking habits intersects with broader social and cultural factors to shape attitudes and behaviors toward alcohol. By addressing these gaps, the analysis could provide a more comprehensive understanding of the complex interplay between social influences and alcohol consumption within the community. | Revised as suggested. |
| The Multifaceted Impact of Alcohol on Life's Dimensions:  The analysis of findings under this theme effectively captures the multifaceted impact of alcohol consumption on various dimensions of life, including health, relationships, work, and financial stability. However, there is a potential weakness in the analysis concerning the depth of exploration into the underlying factors contributing to these impacts and the complexities of their interplay. While the narratives provide valuable insights into individuals' experiences and perceptions, there is a lack of discussion on broader societal factors that may exacerbate or mitigate these effects. For example, the analysis could delve deeper into the structural determinants of alcohol-related harm, such as socioeconomic disparities, access to healthcare, and cultural norms surrounding alcohol use. Additionally, while individual anecdotes shed light on the personal consequences of alcohol consumption, there is limited examination of systemic issues, such as alcohol marketing practices, regulatory policies, and healthcare interventions, which may influence patterns of consumption and associated outcomes. By incorporating a more comprehensive analysis of these contextual factors, the discussion could offer a richer understanding of the complex dynamics underlying the impact of alcohol on individuals and communities. | Revised as suggested. The exploration of the impacts of alcohol was not extensive from the get go, this will be addressed in the limitations and strengths section. |
| Discussion:  While the discussion effectively synthesizes the findings of the study with existing literature, it falls short in explicitly demonstrating the uniqueness of the findings compared to previous studies. Although it references broader trends and insights from prior research, it lacks a clear delineation of how the current study's findings diverge or contribute novel insights to the existing body of knowledge. To strengthen the discussion, it would be beneficial to explicitly highlight any novel findings or unique aspects uncovered by the study that distinguish it from previous research. This could involve identifying specific nuances or patterns in alcohol consumption behaviors within the Sabah community that have not been extensively documented in prior studies. By emphasizing the distinctiveness of the findings, the discussion could further underscore the significance of the study and its contribution to advancing understanding in the field of alcohol consumption research. | Revised as suggested to demonstrate the uniqueness of the study. |
| Conclusion:  The conclusion effectively summarizes the key findings and emphasizes the need for a holistic approach to addressing the complex dynamics of alcohol consumption in the Sabah community. However, one potential weakness is the lack of specific recommendations or actionable steps for addressing the identified challenges. While the conclusion highlights the importance of community-based solutions and awareness-raising efforts, it would be strengthened by providing more concrete suggestions for interventions or policies that could be implemented to address the evolving patterns of alcohol consumption. Additionally, the conclusion could benefit from a brief reflection on the implications of the study's findings for future research or practice in the field of alcohol misuse prevention and intervention. By incorporating these elements, the conclusion would offer a more comprehensive and actionable framework for addressing the complexities of alcohol consumption in the Sabah community. | Revised as suggested. |
