# Supplementary material for: Gendered narratives and cultural shifts: A qualitative study on decadal changes in community alcohol consumption
Source: PLoS One. 2024 Dec 2;19(12):e0309378. doi: 10.1371/journal.pone.0309378 (PMC11611190; doi:10.1371/journal.pone.0309378)
Supplement: Attachment — Submitted filename: Reviewer Comment Checklist 3.docx [file pone.0309378.s005.docx]

**Reviewer’s Comments**

Reviewer 4

| Comments | Remarks |
| --- | --- |
| Abstract:  The abstract needs a major revision to ensure consistency with the research title and to provide a comprehensive overview of the study. Here are the suggested revisions: Clearly articulate specific gendered narratives related to alcohol consumption and how these have evolved over the decade. Provide examples or themes from the qualitative data to illustrate these narratives. Elaborate on the specific cultural transformations that have influenced changes in drinking habits. Explain how these cultural shifts interact with gender dynamics. Describe the qualitative approach in more detail, including the analytical framework used to interpret the focus group interviews. This will help readers understand how the data was gathered and analyzed. Emphasize the decadal aspect by providing a comparative analysis of past and present alcohol consumption patterns. Highlight key differences and trends observed over the ten-year period. Link the economic consequences of alcohol use back to the gendered and cultural context. Explain how these economic impacts are influenced by the changes in gender roles and cultural practices. Discuss the implications of the findings for public health policies and interventions. Emphasize the need for gender-sensitive and culturally appropriate strategies to address alcohol misuse. Also need to write a short sentence about the theoretical framework use in this study to make readers well interested about this research. | The suggestions were addressed and highlighted for reference. |
| Introduction:  However, the section has notable weaknesses. Key terms like “gender differences,” “cultural dynamics,” and “alcohol misuse” are not clearly defined, leading to potential ambiguity. Additionally, while the introduction mentions a literature review and theoretical framework, it fails to provide an overview of the theoretical perspective that will be used. Crucially, the decadal changes in alcohol consumption patterns—a central aspect of the research title-are not emphasized, which weakens the connection to the study’s temporal scope. Furthermore, the study’s specific objectives are not explicitly stated, resulting in a lack of focus. To ensure the introduction aligns strongly with the research title “Gendered Narratives and Cultural Shifts: A Qualitative Study on Decadal Changes in Community Alcohol Consumption,” it requires major revision. This should include defining key terms, summarizing the theoretical framework, emphasizing decadal changes, and clearly outlining the specific research objectives.  Moreover, the introduction section partially highlights the research problem that necessitates this study, but it could be improved for clarity and impact. It discusses the cultural heritage of alcohol consumption in Sabah and the shift towards misuse, indicating a rising incidence of alcohol-related issues such as domestic violence, absenteeism, motor accidents, and physical confrontations. This sets up a context for the research problem but could be more explicit in stating the specific gap in understanding or knowledge that the study aims to address. The importance of the study and its beneficiaries are somewhat implied but not explicitly detailed. The introduction mentions the significance of alcohol in cultural practices and the concerning trends of misuse, suggesting that understanding these issues is crucial. However, it does not clearly illustrate why this study is important or who will benefit directly from the findings. It could benefit from a more explicit statement on the practical implications of the research for public health officials, policymakers, and the local community. The contribution of this study to the broader realm of knowledge is suggested but not fully articulated. It touches on the need for culturally sensitive interventions and the study’s aim to explore the complex relationship between tradition and alcohol-related issues. However, it would be stronger if it explicitly stated how this research fills existing gaps, adds to the academic discourse, or informs future studies and interventions in similar cultural settings. | The suggestions were addressed and highlighted for reference. |
| Literature Review:  The literature review in this section provides a comprehensive overview of existing studies on alcohol consumption in Sabah, Malaysia, focusing on sociocultural, economic, and demographic factors. It effectively integrates various studies to highlight the complexity of alcohol use in the region. However, there are several major shortcomings that need to be addressed.  One critical aspect lacking in the review is the synthesis and integration of findings across studies. While multiple studies are discussed, they are presented in isolation without a concerted effort to connect their findings into a cohesive narrative. This fragmented approach impedes the development of a cumulative understanding of alcohol consumption patterns in Sabah, which is crucial for advancing knowledge in this field. Despite acknowledging the cultural significance of alcohol in Sabah, the review does not deeply explore the unique cultural beliefs and practices that influence alcohol consumption. There is a noticeable gap in understanding how these cultural factors interact with other determinants such as religion or economic conditions to shape drinking behaviors. A more nuanced exploration of these cultural specificities would provide critical insights into the complex dynamics at play and enhance the relevance of the research findings to the local context.  In terms of addressing research gaps, although the review identifies the need for a comprehensive exploration of cultural and gender dynamics in alcohol consumption, it falls short in explicitly detailing how the current study fills these gaps. There is a gap between the identified shortcomings in the literature and the explicit alignment of research objectives to address these deficiencies. A stronger emphasis on how the current study contributes to filling these gaps would enhance the clarity and purpose of the research endeavor.  Regarding gender aspects, while the title emphasizes “Gendered Narratives and Cultural Shifts,” the review does not adequately assess the breadth and depth of literature specifically addressing gender dynamics in alcohol consumption in Sabah. There is insufficient evidence of a focused exploration of how gender roles and perceptions influence alcohol use patterns within the reviewed studies. This gap diminishes the alignment of the literature review with the study’s explicit focus on gendered narratives, suggesting a need for more targeted exploration and synthesis of gender-related findings in future research. | The suggestions were addressed and highlighted for reference. |
| Methodology:  Reflexivity and Researcher Bias:  Weakness: The section on reflexivity acknowledges the main researcher’s biases and challenges in maintaining neutrality but lacks explicit detail on how these biases were mitigated throughout the study.  Suggestions: Expand on strategies used to minimize researcher bias, such as peer debriefing, maintaining reflective diaries, and continuous dialogue with supervisors. Detail how biases were identified and managed during data collection and analysis.  Ethical Considerations:  Weakness: Although measures to protect participant identities are mentioned, there's no discussion on how confidentiality was ensured during data storage and analysis phases.  Suggestions: Include details on data anonymization procedures and secure data storage practices to maintain confidentiality. Discuss any ethical challenges encountered during the study and how they were addressed.  Data Analysis:  Weakness: The use of ChatGPT for data organization and thematic analysis is mentioned, but there’s limited discussion on its role beyond organization and retrieval.  Suggestions: Provide more insight into how ChatGPT specifically contributed to the thematic analysis process. Discuss its limitations and strengths in relation to qualitative data analysis and interpretation.  Overall Revision Guidance:  Integration and Clarity: Ensure each subsection of the methodology is tightly integrated to provide a clear, sequential flow of the research process.  Detail and Justification: Expand on methodological choices with clear justifications based on the study's objectives and the context of Sabah’s cultural dynamics.  Transparency and Reflection: Emphasize transparency in reporting methods and reflect critically on limitations and challenges encountered.  Ethical Rigor: Strengthen discussions on ethical considerations and participant protections throughout all stages of the research. | The suggestions were addressed and highlighted for reference. |
| Results:  The Results section provides a comprehensive exploration of themes emerging from the study on alcohol consumption in Sabah, Malaysia. Here are identified weaknesses and suggestions for improvement:  Clarity and Organization:  Weakness: While each theme is discussed, there’s a need for clearer transitions between themes and sub-themes to enhance readability and coherence.  Suggestions: Consider structuring each theme with subheadings to delineate different aspects (e.g., Tradition and the Shifting Role of Alcohol, The Double-Edged Sword of Visibility and Accessibility, Social Influences and Evolving Gender Norms in Alcohol Consumption, The Multifaceted Impact of Alcohol Consumption). This will aid readers in navigating through complex information more easily.  Depth and Detail in Analysis:  Weakness: Some themes, such as the impact of alcohol on health and relationships, are extensively covered, while others, like the cultural and traditional aspects, could benefit from more detailed exploration.  Suggestions: Ensure each theme is analyzed comprehensively by including diverse perspectives and examples that reflect the richness of participant responses. Expand on cultural nuances and historical contexts where relevant to deepen understanding.  Integration of Data:  Weakness: The integration of participant quotes and specific examples is uneven across themes, impacting the overall robustness of the findings.  Suggestions: Consistently integrate participant quotes and examples throughout each theme to illustrate key points and enhance the credibility of findings. Ensure that quotes are contextually relevant and provide sufficient detail to support thematic interpretations. | The suggestions were addressed and highlighted for reference. |
| Discussion:  The Discussion section provides a thorough analysis of the study findings on alcohol consumption in Sabah, Malaysia, yet there are areas that could benefit from revision and enhancement:  Integration of Literature and Findings:  Weakness: While the discussion references previous literature and aligns findings with existing research, there could be more explicit connections drawn between specific findings and the broader theoretical frameworks or empirical studies cited.  Suggestions: Strengthen the discussion by directly linking each thematic finding to relevant literature on cultural traditions, socioeconomic influences, gender dynamics, and health impacts. Discuss how these findings contribute to or challenge existing theories or knowledge gaps in the field.  Depth of Analysis:  Weakness: Some aspects of the discussion, such as the impact of the COVID-19 pandemic on alcohol consumption patterns, could benefit from deeper analysis and exploration of contrasting perspectives or unexpected findings.  Suggestions: Provide a nuanced analysis of how pandemic-related restrictions affected different demographic groups and geographic regions within Sabah. Consider discussing implications beyond immediate consumption patterns, such as long-term behavioral changes or resilience strategies observed.  Contextualization and Generalization:  Weakness: While local traditions and socioeconomic factors are discussed, there is a need for clearer contextualization of findings within the broader Malaysian or global context of alcohol consumption trends.  Suggestions: Discuss how findings in Sabah contribute to understanding regional variations in alcohol consumption across Malaysia or Southeast Asia. Address limitations in generalizing findings beyond the studied communities, emphasizing the unique cultural and geographic factors influencing Sabahan drinking behaviors.  Implications and Recommendations:  Weakness: The discussion could be strengthened by providing more explicit recommendations for policy interventions or community-based strategies based on study findings.  Suggestions: Outline specific recommendations for health interventions, community programs, or policy changes aimed at addressing alcohol-related health disparities, promoting responsible drinking practices, and supporting cultural preservation efforts. Discuss how these recommendations align with local priorities and community needs.  Overall Revision Guidance:  Synthesis of Findings: Ensure each thematic finding is thoroughly discussed in relation to existing literature, theories, and empirical evidence, highlighting both confirmatory and novel aspects of the study.  Critical Analysis: Provide a critical analysis of findings, discussing contradictions or unexpected results in relation to theoretical frameworks or practical implications.  Contextualization: Situate findings within the broader socio-cultural, economic, and health contexts to enhance understanding of alcohol consumption patterns in Sabah.  Recommendations: Offer clear and actionable recommendations for stakeholders, policymakers, and community leaders based on study findings, emphasizing evidence-based strategies to address identified challenges. | The suggestions were addressed and highlighted for reference. |
| Conclusion:  A strong conclusion typically summarizes the main points discussed in the paper, reaffirms the significance of the research findings in relation to the broader literature, and clearly outlines the practical implications and recommendations derived from the study. In this case, while the Conclusion touches upon the complexities of alcohol consumption patterns and acknowledges the socio-cultural factors at play, it could benefit from a more structured approach to summarizing how these factors interact and influence alcohol use in the Sabah community. Moreover, there’s a need for a clearer connection between the findings and the recommended actions or interventions proposed in the Recommendations section. This would strengthen the overall coherence of the paper and reinforce the relevance of the study’s findings to both academic discourse and practical applications in community health and policy development. Therefore, improving the clarity and coherence of the Conclusion section by providing a concise synthesis of findings and recommendations would enhance the overall impact and scholarly rigor of the study | The suggestions were addressed and highlighted for reference. |
